# Evaluation of influences of forest cover change on landslides by comparing rainfall-induced landslides in Japanese artificial forests with different ages

**DOI:** 10.1038/s41598-023-41539-x

**Published:** 2023-08-31

**Authors:** Tadamichi Sato, Yoh Katsuki, Yasuhiro Shuin

**Affiliations:** 1 Graduate School of Bioresource and Bioenvironmental Sciences, Kyushu University, Fukuoka, Japan; 2 Faculty of Agriculture, Kyushu University, Fukuoka, Japan

**Keywords:** Natural hazards, Hydrology, Civil engineering

## Abstract

In this study, we evaluated the influence of forest cover changes on rainfall-induced shallow landslides by comparing two shallow landslides and debris flows that occurred on plantation forests of different ages in Japan: the Kake disaster in 1988 and the Asakura disaster in 2017. At Kake, the trees ranged in age from 10 to 30 years, whereas at Asakura the trees were over 40 years old. The rainfall characteristics that triggered each landslide were estimated using a three-layer tank model, and the results, as well as the volume of driftwood produced by the landslides, were then compared. Both landslides occurred when the first tank storage layer value, corresponding to the temporal variation in groundwater level in the shallow soil layer, exceeded its previous maximum. The return period of this value at the time of the landslides was 3.0-fold higher in the more mature forests of Asakura than in the young forests of Kake. The upper limit of driftwood volume was 30-fold higher in Asakura than in Kake. Our findings indicated that shallow landslides and debris flows become increasingly rare as forests mature; however, the large volume of driftwood produced by landslides in mature forests may cause substantial damage when extreme rainfall events exceed the landslide resistance of those forests. These insights may be applied to effective landslide risk management.

## Introduction

Rainfall-induced landslides occur due to complex interactions between inducing (rainfall characteristics) and intrinsic (geomorphology, geology, vegetation, etc.) factors and have substantial social impacts^1,2^. Rainfall characteristics, i.e., intensity, duration, and antecedent rainfall are strongly linked to triggering mechanisms and timing of different types of landslides^1,3^. On the other hand, the slope shape and gradient in geomorphology affect slope stability^4,5^. Similarly, geological structures, weathering profiles, and soil formation in geology affect the occurrence of landslides^5–7^. Vegetation cover controls the magnitude and rate of shallow landslides induced by heavy rainfall^8,9^. Besides, human activities such as forest harvesting, quarrying stones, and land use change impact natural factors and thereby alter the occurrence of landslides^8–11^. To mitigate the damage caused by landslides, it is necessary to understand the relationship between landslides and the factors that trigger them, including both inducing and intrinsic factors.

Forest cover is an important intrinsic factor influencing landslides. Forest cover reduces the likelihood of shallow landslides because tree roots increase shearing resistance^12,13^, but this protective function varies with changes in forest cover (e.g., clear-cutting and subsequent afforestation)^10,14–19^. Imaizumi et al.^10^ examined the effects of clear-cutting on landslides and demonstrated that temporal variations in landslide occurrence were explained by the decline and recovery of root strength. However, the effects of clear-cutting on hydrogeomorphological processes cannot be estimated based only on the time elapsed since cutting^16^, and it is important to consider rainfall characteristics when assessing the effects of changes in forest cover on landslides.

Numerous studies have explored the relationship between rainfall characteristics and landslides. Rainfall characteristics are estimated using empirical methods that consider rainfall intensity and duration^20,21^, conceptual models of infiltration^22–25^, and process-based models that capture the effects of topography, vegetation, and other intrinsic factors^26–28^.

The three-layer tank model^23^ is a conceptual model that describes the relationship between rainfall characteristics and landslides while accounting for the influence of antecedent rainfall^25^. Sato and Shuin^25^ examined shallow and deep-seated landslides in Mie prefecture using this model and demonstrated that temporal variations in the first and third tank storage layer values were correlated with shallow and deep-seated landslides, respectively. They also reported that the influence of intrinsic factors can be estimated by comparing the rainfall characteristics that caused the landslide using a three-layer tank model with the same parameters^25^. Although some studies have evaluated the effects of changes in forest cover on landslide occurrence by focusing on thresholds of landslides based on rainfall intensity and duration^16,17,19^, none have explored the links between landslides and forest cover using the three-layer tank model.

In Japan, rainfall-induced landslides varied as forest cover changed^29–32^. Numamoto et al.^29^ investigated reports of deaths and disappearances following sediment-related disasters between the 1940s and 1990s and reported decreased incidences of deaths and disappearances caused by rainfall-induced landslides, which are likely to be attributable to changes in forest maturity. Likewise, Sato and Shuin^30^ examined the impact of changes in forest cover at the national scale in Japan on floods and sediment-related disasters triggered by heavy rainfall and showed that damaged areas by floods and sediment-related disasters decreased as forest cover, particularly artificial forests matured. Furthermore, Tsukamoto^32^ noted that shallow landslides induced by heavy rainfall were infrequent owing to increased forest cover and maturity.

By contrast, forest cover matured and so, larger volumes of driftwood have been produced by landslides in recent years and have increased the damage^33,34^; for example, heavy rainfall triggered landslides in northern Kyushu, Japan on July 5, 2017^34^, triggering the largest volumes of driftwood documented in history^35^. In consequence, addressing driftwood has become an issue in the national policy agenda^36^ and the influence of maturity of forest cover on the driftwood contained in landslides is also required to be evaluated^37^.

Therefore, our objective was to evaluate the influences of changes in forest cover on shallow landslides induced by heavy rainfall, by comparing two landslides that occurred in plantation forests of different ages. A young forest was defined as < 30 years and a mature forest as > 30 years old. Differences in the thresholds of rainfall characteristics that triggered the landslides and in the volume of driftwood produced by the landslides were compared between the two study areas. Features of landslides in the study areas were examined using a three-layer tank model to estimate rainfall characteristics, normalized precipitation, and driftwood volume. The thresholds of rainfall characteristics that triggered the landslides and the resulting driftwood volumes were compared to determine the influence of changes in forest cover on landslides induced by heavy rainfall events. The results of this study provide novel insights into the relationship between changes in forest cover and rainfall-induced landslides, and will contribute to the improvement of landslide risk management strategies.

## Methods

### Study areas and landslides

**Figure 1:**
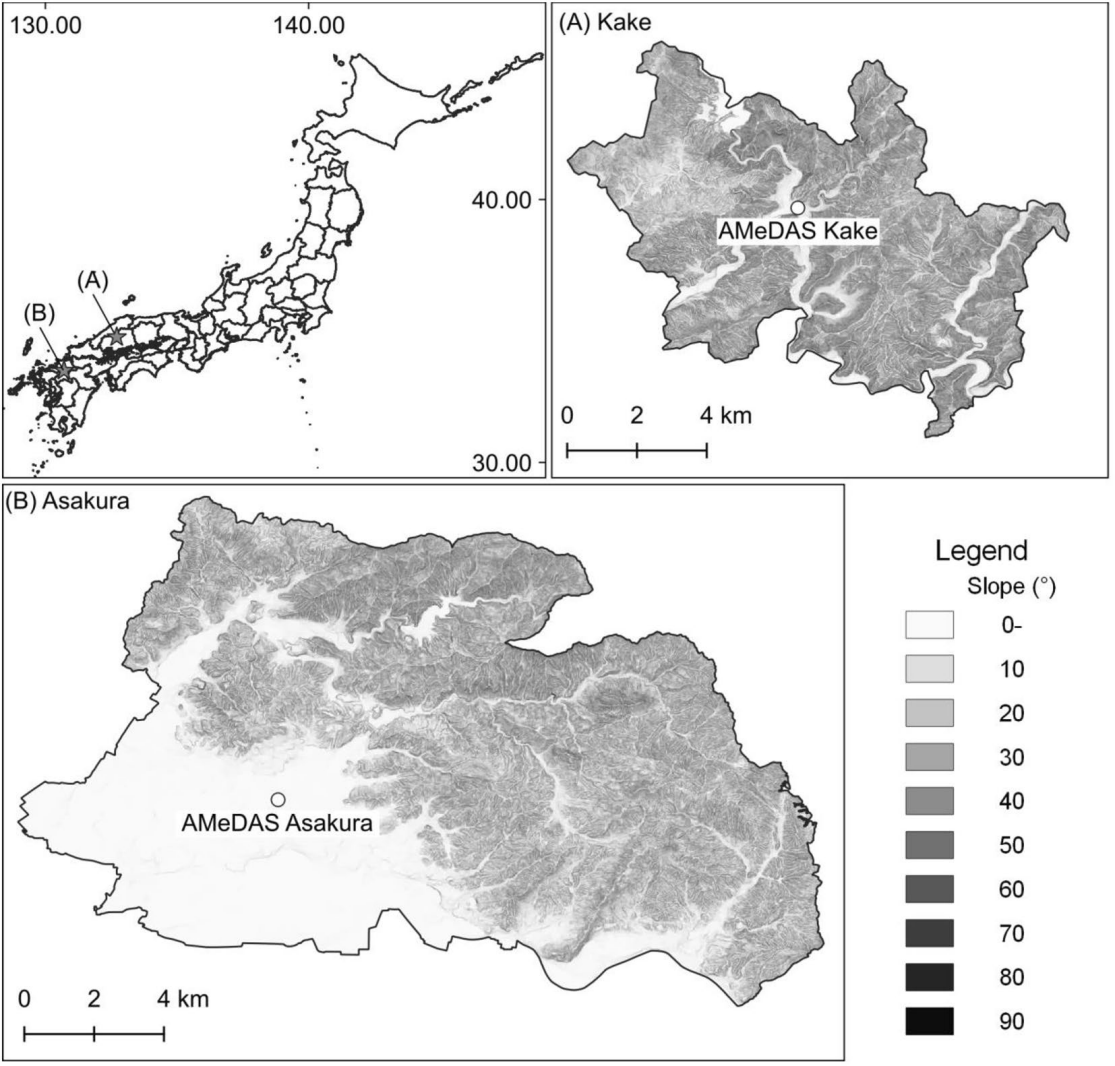
Study area. White circles indicate AMeDAS.

The town of Kake, in Hiroshima Prefecture, and the city of Asakura, in Fukuoka Prefecture (Fig. 1), were selected for the study for several reasons. First, two landslides occurred in plantation forests of different ages: a young forest (10–30 years old) at Kake and a mature forest (> 40 years old) at Asakura^38,39^. In addition, the occurrence of shallow landslides that are affected by forest cover was dominant^34,40^. Both landslides produced substantial amounts of driftwood^35,38^, and the precise timing of the shallow landslides and resulting debris flows was observed in both disasters^40,41^. Finally, geological conditions are comparable between the two sites as both are underlain by granite^34,40^, and the area of steep hillslope with 30 degree or over of slope angle, where shallow landslides is likely to occur^42^, is almost the equal in two study area (Kake is 48.0 km^2^, Asakura is 53.4 km^2^). Thus**,** we selected same types of landslides in areas with similar conditions in geological and topographical to focus on the influences of changes in forest cover.

#### Kake

The town covers an area of 96.1 km^2^ and ranges in elevation from 78.8 m to 997.1 m above sea level (a.s.l.). The mean annual precipitation (1976–2020) is 1859.4 mm, and the geology is dominated by granite, sandstone, and mudstone^43^.

**Figure 2:**
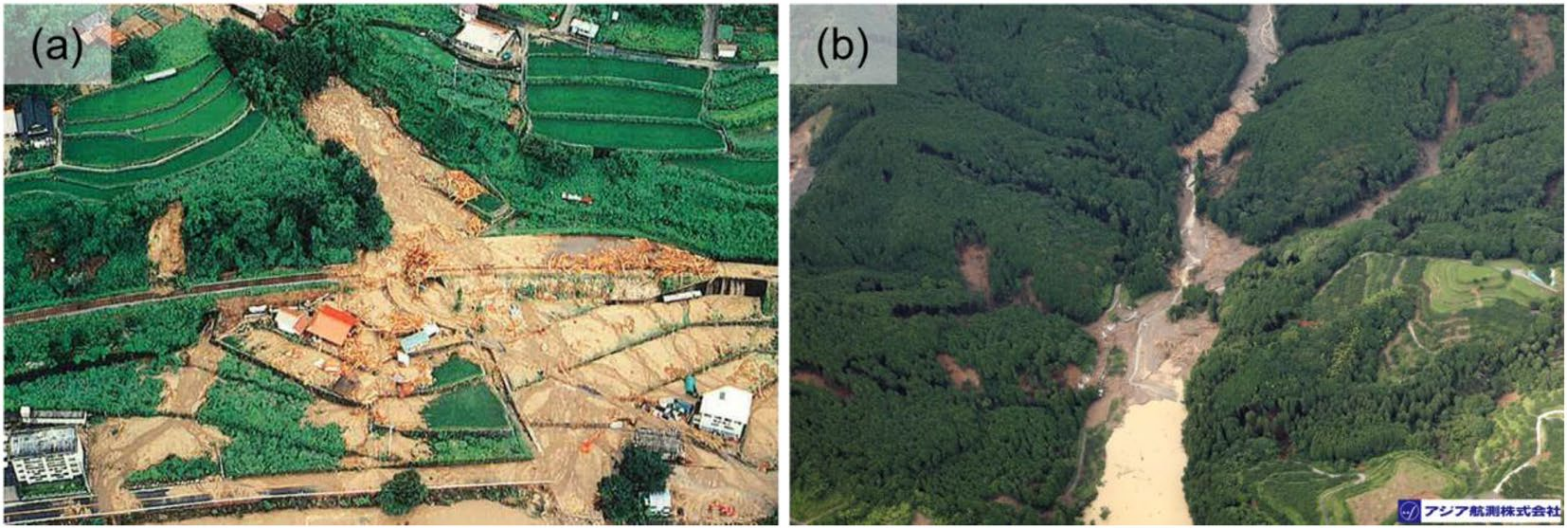
Typical landslides and debris flows, including driftwood in the Kake disaster on July 19, 1998 (**a**)^43^ and the Asakura disaster on July 5, 2017 (**b**) (Photo by Asia Air Survey Co. Ltd.).

A severe rain event in the northwestern part of Hiroshima Prefecture on July 20 and 21, 1988 produced an hourly maximum of 57 mm rainfall and a total of 264 mm rainfall^40^. In Kake, this event triggered shallow landslides and debris flows that included driftwood (Fig. 2a)^38,40, 44^. Landslides occurred in artificial forests of Kake dominated by 10–30-year-old Japanese sugi (*Cryptomeria japonica*) trees^38^. Interviews with residents indicate that the landslide and debris flows occurred between 2:30 p.m. and 3:30 p.m. on July 21^40^.

#### Asakura

Asakura covers an area of 246.7 km^2^ and ranges in elevation from 10.1 m to 932.5 m a.s.l. The mean annual precipitation (1976–2020) is 1934.0 mm. The area is largely underlain by andesite, with some granodiorite, breccia, and sandstone^43^.

A storm that occurred between July 5 and 7, 2017 between Asakura City and Hita City produced hourly and daily maximums of 140 and 800 mm rainfall (Oita prefecture)^34^. Given the mountainous terrain, several landslides and debris flows, which included driftwood, were triggered (Fig. 2b). The geology comprises decomposed granite soil, pelitic schist, and andesite^34^. The landslides occurred near forests comprising > 40-year-old Japanese sugi and hinoki (*Chamaecyparis obtusa*) trees^39^. Interviews with residents indicated that the shallow landslides and debris flows occurred at 3:00 p.m. on July 5^41^.

### Data collection

Hourly rainfall data from 1976 to 2020 were collected by Automated Meteorological Data Acquisition System (AMeDAS) stations at Kake and Asakura to examine rainfall characteristics using the three-layer tank model (Fig. 1). Driftwood volume and catchment area data were obtained from a previous study^38^ and a disaster report^35^. Driftwood was investigated at Kake using aerial photogrammetry and a field survey^38^, and at Asakura using aerial photogrammetry^35^. The average area of the investigated catchments was 0.30 km^2^ (range, 0.01–2.84 km^2^).

### Three-layer tank model

**Figure 3:**
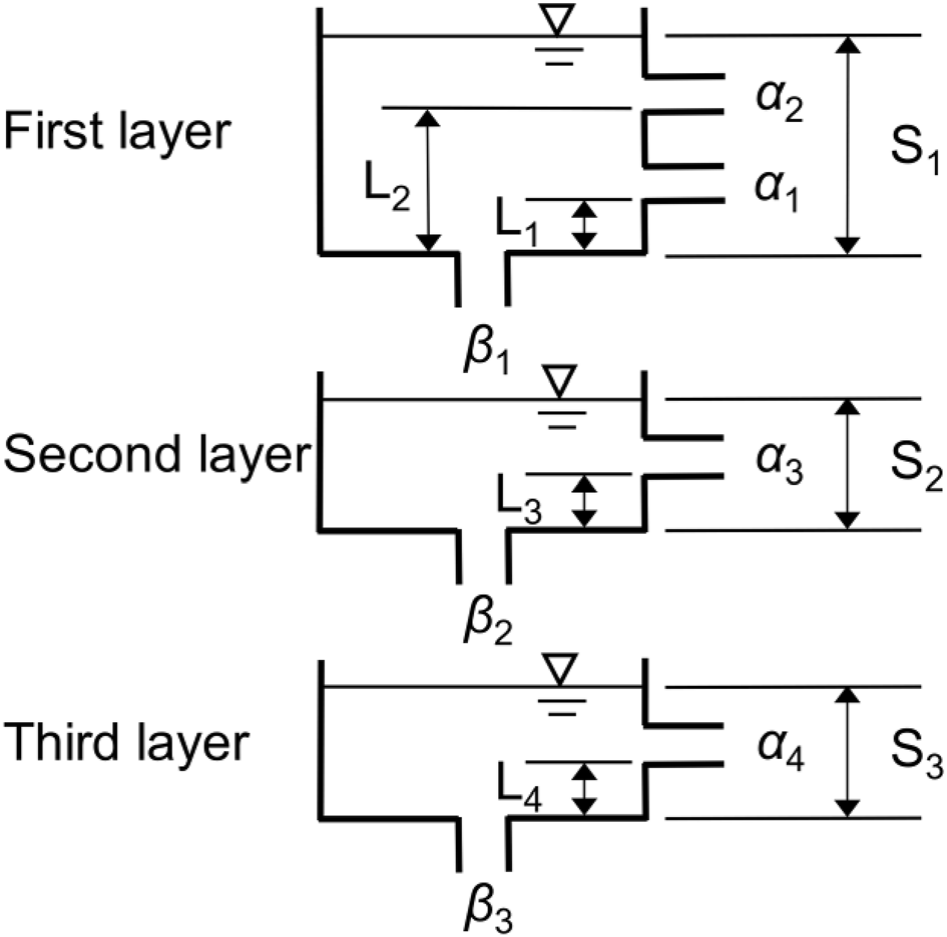
Schematic diagram of the three-layer tank model (Ishihara and Kobatake 1979^23^).

A three-layer tank model was used to estimate the rainfall characteristics that triggered the investigated landslides. The model^23^ consists of three vertically arranged tanks, each with outlets at the side and bottom (Fig. 3), which represent the infiltration–storage process. In the model, temporal variation in the storage values of the upper (first layer), middle (second layer), and lower (third layer) tanks correspond to temporal variation in groundwater levels (pore water pressure) in the upper, middle, and lower soil layers, respectively. Changes in the value of each tank storage layer are therefore assumed to represent changes in the groundwater level of the respective soil layer.

The three-layer tank model is a simple lumped model that yields data only on the average parameters of inherent factors in the watershed^26–28^, in contrast to distribution models, which consider their spatial distribution. The tank model easily calculates long-term groundwater levels while accounting for the influence of antecedent rainfall, and can be used to estimate the rainfall characteristics that trigger landslides^25^. Values for each layer were calculated as follows:

1 $$\begin{array}{l}{S}_{1}\left(t+\Delta t\right)=\left(1-{\beta }_{1}\Delta t\right)\cdot {S}_{1}\left(t\right)-{q}_{1}\left(t\right)\cdot \Delta t+R \end{array}$$

2 $$\begin{array}{l}{S}_{2}\left(t+\Delta t\right)=\left(1-{\beta }_{2}\Delta t\right)\cdot {S}_{2}\left(t\right)-{q}_{2}\left(t\right)\cdot \Delta t+{\beta }_{1}\cdot {S}_{1}\left(t\right)\cdot \Delta t \end{array}$$

3 $$\begin{array}{l}{S}_{3}\left(t+\Delta t\right)=\left(1-{\beta }_{3}\Delta t\right)\cdot {S}_{3}\left(t\right)-{q}_{3}\left(t\right)\cdot \Delta t+{\beta }_{2}\cdot {S}_{2}\left(t\right)\cdot \Delta t \end{array}$$

where *S*_1_–*S*_3_ represent tank storage layer value (mm), *β*_1_– *β*_3_ are coefficients of permeability (h^–1^), and *q*_1_–*q*_3_ are the outflows from each tank. The time step (*Δt*) is 10 min; *R* represents rainfall per 10-min period (mm/10 min) and was obtained by dividing hourly rainfall data by 6. Discharge volumes from tank outflows were calculated as follows:

4 $$\begin{array}{l}{q}_{1}\left(t\right)={\alpha }_{1}\left\{{S}_{1}\left(t\right)-{L}_{1}\right\}+{\alpha }_{2}\left\{{S}_{1}\left(t\right)-{L}_{2}\right\} \end{array}$$

5 $$\begin{array}{l}{q}_{2}\left(t\right)={\alpha }_{3}\left\{{S}_{2}\left(t\right)-{L}_{3}\right\} \end{array}$$

6 $$\begin{array}{l}{q}_{3}\left(t\right)={\alpha }_{4}\left\{{S}_{3}\left(t\right)-{L}_{4}\right\} \end{array}$$

where *α*_1_–*α*_4_ are the outflow coefficients (h^–1^), and *L*_1_–*L*_4_ are the outflow heights (mm). Ishihara and Kobatake^23^ performed a runoff analysis in five actual basins with different geology (volcanic rock, granite, palaeozoic, tertiary, and quaternary) in Japan and determined the tank model parameters corresponding to the geology. In this study, tank model parameters for a granitic substrate (Table 1) were used because granite is the dominant rock in the study areas^34,40^. To reflect on the influences of inherent factors within study areas, determining the appropriate parameters through runoff analysis would be preferable. However, a three-layer tank model with uniform parameters has been employed for landslide early warning systems across Japan, demonstrating its effectiveness^24^. Additionally, a comparison of rainfall characteristics that trigger landslides using the three-layer tank model with common parameters enables estimating the effect of inherent factors^25^. Hence, this study adopted the three-layer tank model with the same parameters to consistently evaluate rainfall characteristics.

**Table 1:** Tank model parameters (Ishihara and Kobatake 1979^23^).

Tank	First layer		Second layer		Third layer	
Outflow height (mm)	*L*_1_	15.0	*L*_3_	15.0	*L*_4_	15.0
	*L*_2_	60.0				
Outflow coefficient (1/h)	*α*_1_	0.10	*α*_3_	0.05	*α*_4_	0.01
	*α*_2_	0.15				
Coefficient of permeability (1/h)	*β*_1_	0.12	*β*_3_	0.05	*β*_3_	0.01

### Extreme value analysis

Landslide occurrence is more strongly affected by the regional occurrence probabilities of rainfall events of a certain magnitude than by rainfall amount^25^. Accordingly, rainfall amount is often normalized to examine the relationship between the occurrence of landslides and rainfall characteristics^21,25,45^. In this study, we converted each tank storage layer value to the return period (RP) to compare the characteristics of the two rainfalls. We used the parameters of the generalized extreme value distribution (GEV)^46^, estimated using the L-moment method^47^, to calculate the RP of each tank storage layer value between 1976 and 2020. The RP is calculated as follows:

7 $$\begin{array}{l}RP=\frac{1}{1-F\left(x\right)} \end{array}$$

8 $$\begin{array}{l}F\left(x\right)=exp\left\{{-\left(1-k\frac{x-c}{a}\right)}^\frac{1}{k}\right\} for k\ne 0 \end{array}$$

where *F*(x) is the non-exceedance probability of GEV, *k* is the shape parameter, *c* is the scale parameter, and *a* is the location parameter. Parameters of the GEV were calculated as follows:

9–11 $$\begin{array}{l}\left\{\begin{array}{l}k=7.8590d+2.9554{d}^{2}\\ a=\frac{k{\lambda }_{2}}{\left(1-{2}^{-k}\right)\Gamma \left(1+k\right)}\\ c={\lambda }_{1}-\frac{a}{k}\left\{1-\Gamma \left(1+k\right)\right\}\end{array}\right. \end{array}$$

12 $$\begin{array}{l}d=\frac{{2\lambda }_{2}}{{\lambda }_{3}+{3\lambda }_{2}}-\frac{\mathrm{ln}\left(2\right)}{\mathrm{ln}\left(3\right)} \end{array}$$

where *λ*_1-3_ are sample L-moments, and *Γ* was gamma function. *λ*_1-3_ are given as:

13–15 $$\begin{array}{l}\left\{\begin{array}{l}{\lambda }_{1}={\beta }_{0}=\frac{1}{N}\sum_{j=1}^{N}{x}_{\left(j\right)}\\ {\lambda }_{2}={\beta }_{1}=\frac{1}{N\left(N-1\right)}\sum_{j=1}^{N}\left(j-1\right){x}_{\left(j\right)}\\ {\lambda }_{3}={\beta }_{2}=\frac{1}{N\left(N-1\right)\left(N-2\right)}\sum_{j=1}^{N}\left(j-1\right)\left(j-2\right){x}_{\left(j\right)}\end{array}\right. \end{array}$$

where *x*_(*j*)_ is the *j*-th value from the smallest when the sample is arranged in increasing order.

### Evaluation of driftwood volume produced by landslides

The relationship between driftwood volume per unit catchment area and catchment area was calculated as described previously^38,48^:

16 $$\begin{array}{l}{V}_{ga}=b\times {A}^{-1} \end{array}$$

where *V*_*ga*_ is the driftwood volume per unit catchment area, *A* is the catchment area, and *b* is a constant. Driftwood volumes per unit catchment area in the two study areas were compared based on their 100^th^ and 50^th^ percentiles.

## Results and discussion

### Comparison of rainfall characteristics

**Figure 4:**
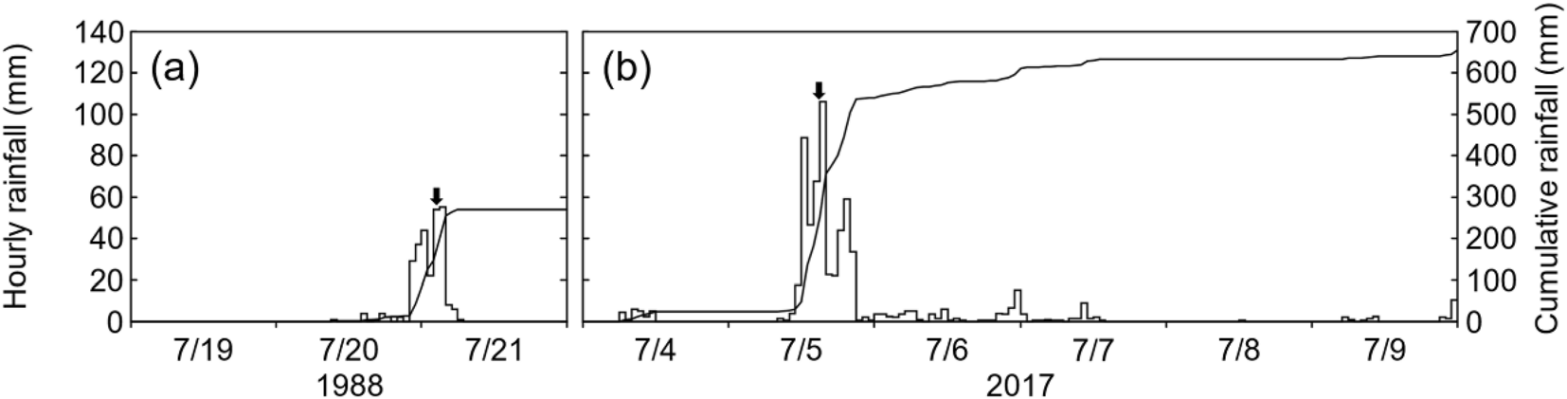
Rainfall events that caused shallow landslides and debris flows in Kake (**a**) and Asakura (**b**). Black arrows indicate the timing of the landslides.

Characteristics of the focal rainfall events are shown in Fig. 4. Following Osanai et al.^24^, rainfall events were considered distinct when separated by a 24-h rain-free period. In Kake, the maximum hourly rainfall was 55.0 mm, and the cumulative rainfall was 291.0 mm (Fig. 4a). The maximum hourly rainfall in Asakura was 106.0 mm, and the cumulative rainfall was 654.5 mm (Fig. 4b). The amount of rain that triggered the two landslides differed; however, both landslides occurred during the rainfall peak. These results agree with Iverson^49^, who demonstrated that shallow landslides and debris flows are triggered by short-duration, high-intensity rainfall events.

**Figure 5:**
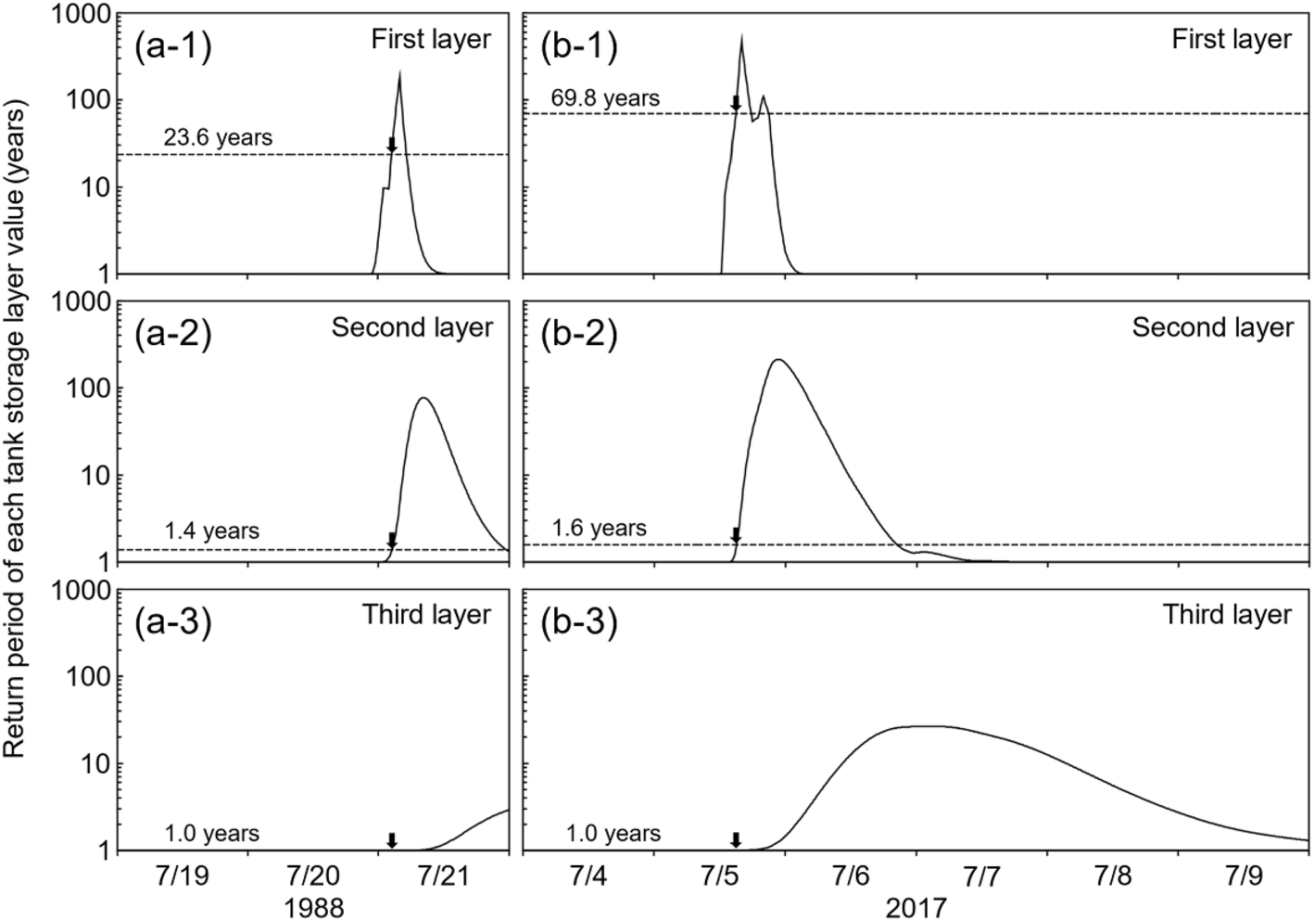
Temporal variations in the RP of each tank storage layer value in Kake (**a1–3**) and Asakura (**b1–3**). Black arrows indicate the timing of the landslides.

**Figure 6:**
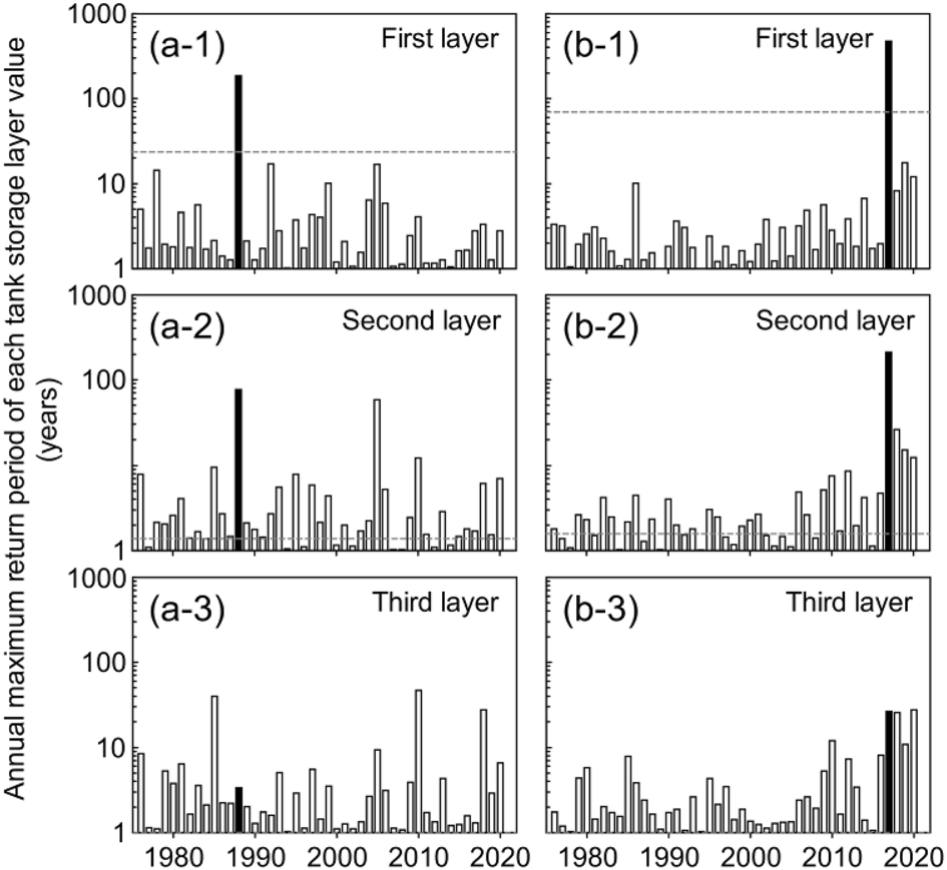
Annual maximum return periods of each tank storage layer from 1976 to 2020. Black bar indicates the year of landslide occurrence; gray dotted line indicates the return period at the time of each landslide.

The temporal variations in the RP of the tank storage layer values are shown in Fig. 5. In the Kake disaster, the RP of the first, second, and third tank storage layer values were 23.6 years, 1.4 years, and 1.0 year, respectively, at the time of the landslide (Fig. 5a1–a3). In the Asakura disaster, the respective values were 69.8 years, 1.6 years, and 1.0 year at the time of the landslide (Fig. 5b1–b3). Both landslides occurred when the RP of the first tank storage layer was higher than its previous maximum (Fig. 6a-1, b-1), and the RPs of the second and third tank storage layers were below their previous maxima (Fig. 6a-2, a-3, b-2, b-3). Sato and Shuin^25^ also examined the relationship between landslides and rainfall characteristics using a three-layer tank model and similarly demonstrated that shallow landslides occurred when the RP of the first tank storage layer exceeded its previous maximum. In addition, the tank model used in this study obviously separated rainfall characteristics triggering landslides from the others because the RP of the first tank storage layer value in years when no landslides occurred, never exceeded it at the landslide occurrences throughout 45 years (Fig. 6a-1, b-1). Together, these results show that the first tank storage layer value represents a rainfall characteristic that is correlated with shallow landslide occurrence, and it was valid to employ the tank model with common parameters for estimating rainfall characteristics triggering landslides.

### Comparison of driftwood volume per unit of catchment

**Figure 7:**
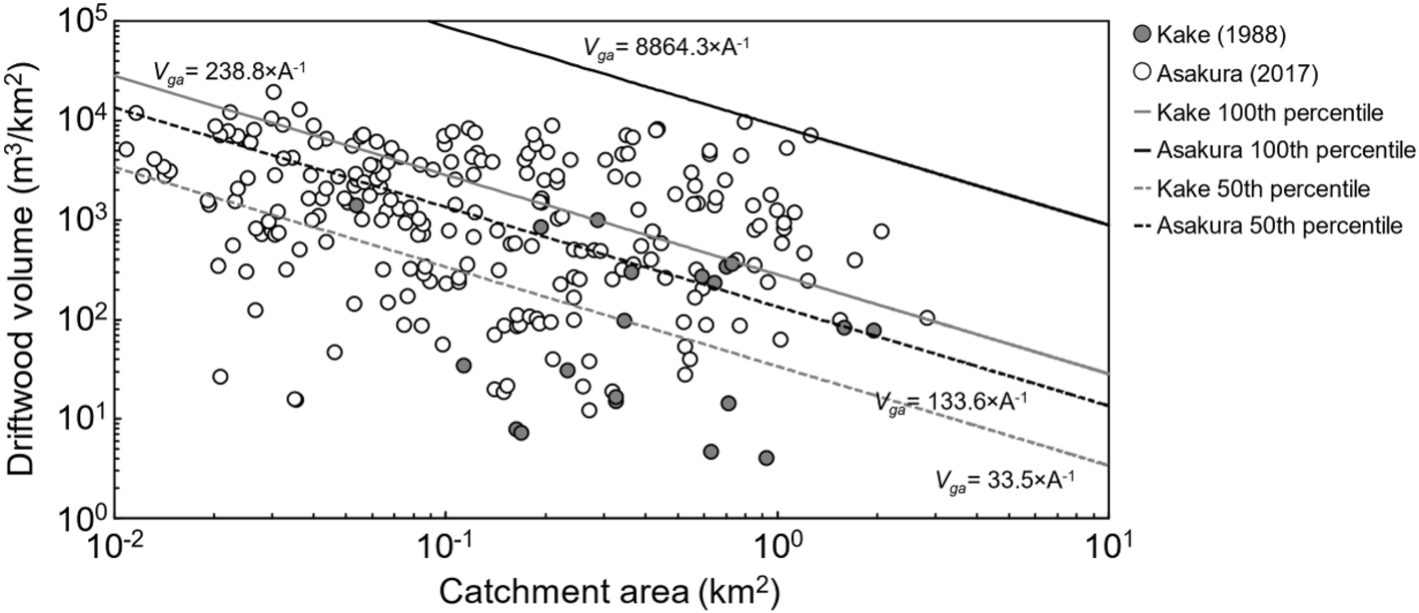
Relationship between driftwood volume per unit catchment area (black circles) and catchment area at Kake (gray circles) and Asakura (white circles). Solid and dotted lines indicate the 100th and 50th percentiles, respectively.

Driftwood volume per unit catchment area triggered by a rainfall event at each site is shown in Fig. 7. Gray and white circles indicate Driftwood volume per unit catchment area against each catchment area in the Kake disaster and in the Asakura disaster, respectively. As shown in Fig. 7, driftwood volume per unit catchment area tended to decrease as an increase in catchment area. Comiti et al.^48^ obtained data from the literature on driftwood volume (per unit catchment area) and catchment area and examined their relationship. They showed that the volume per unit catchment area decreases as the catchment area increases, echoing the trends in our results (Fig. 7).

The 100th (50th) percentile of driftwood volume per unit catchment area was 30 (4) times higher at Asakura than at Kake (Fig. 7). Ministry of Land, Infrastructure, Transport and Tourism^35^ compared driftwood volume between the Asakura disaster and other disasters and demonstrated that the volume of driftwood in the Akatani River during the Asakura disaster was 20 times higher than normal; these data included driftwood data obtained after the Kake disaster. Thus, the relationship between the upper limits of the Asakura and Kake disasters is valid.

### Effect of different forest cover on landslides induced by heavy rainfall

Both landslides occurred during the rainfall peak (Fig. 4) and were correlated with the first tank storage layer value (Figs. 5, 6); however, the RP of this value was 3.0 times higher in Asakura than in Kake. These results indicate that thresholds of landslides differ; these differences are likely attributable to differences in the maturity of forest cover because the geological and topographical features is no major difference between the two study areas. In the Kake disaster, the landslide occurred in plantation forests dominated by 10–30-year-old Japanese Sugi^38^. In the Asakura disaster, large (> 40-year-old) Japanese Sugi and Hinoki trees were located near landslide areas^39^. As forests mature, increased forest cover results in increased resistance to shallow landslides^10,14–19^. For example, Imaizumi et al.^10^ examined the effects of forest harvesting on the frequency of landslides and debris flows in the Sanko catchment, Japan and reported that the direct impacts of clearcutting on landslide occurrence were greatest in stands clearcut during the previous 10 years, and that these effects progressively weakened up to 25 years after harvesting. Therefore, the difference in forest cover maturity corresponded to the difference in the RP of the first tank layer value at the timing of landslides in two disasters, and our results corroborate the results of previous studies.

**Figure 8:**
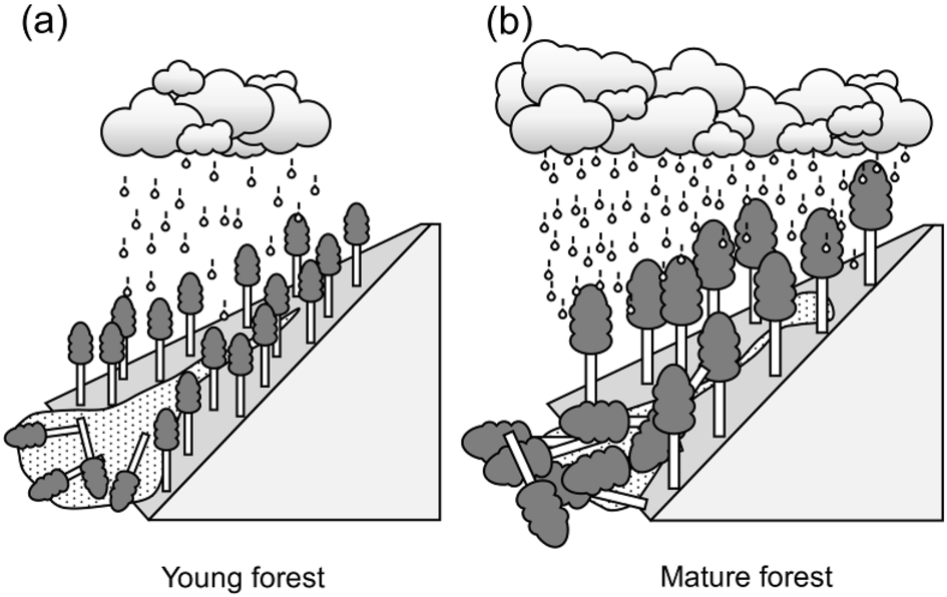
Conceptual diagram of changes in landslide including driftwood as forest cover matures. In young forests, shallow landslides are often caused by heavy rainfall and so, a small amount of driftwood is produced (**a**). In mature forests, shallow landslides are infrequent because the resistance of forests against shallow landslides increases; however, a large amount of driftwood is produced when landslides are triggered by extreme rainfall that exceeds the resistance of forests against shallow landslides (**b**).

Nevertheless, shallow landslides also occurred in mature forests in Asakura, due to an extreme rainfall event whose unusual intensity was related to climate change^50^. As a result, the amount of driftwood volume produced was 30-fold higher at Asakura than at Kake (Fig. 7), such that the amount of damage induced was also higher^34–36^. Thus, our results indicate that although shallow landslide occurrence declines as forests mature, large volumes of driftwood may be produced by landslides when extreme rainfall events exceed the protective function of those forests (Fig. 8), resulting in severe economic and social impacts.

Numerous studies have shown that forest cover improves slope stability^13^, while forest maturity reduces the occurrence of shallow landslides^10,15,18^. However, the adverse effects of changes in forest cover on shallow landslides have not been fully comprehended^37,51^. Our case study of landslides in artificial forests in Japan demonstrated both positive (i.e., increasing thresholds of landslide occurrences; Fig. 5) and negative (i.e., producing large volume of driftwood; Fig. 7) influences of forest cover maturity on rainfall-induced landslides. Although further investigation is required (e.g., considering the spatial distribution of precipitation, forest cover, landslides, and driftwood volume), our findings provide a better understanding of the relationship between forest cover changes and rainfall-induced shallow landslides and will contribute to developing more effective landslide management measures.

## Conclusion

The influences of forest cover changes on shallow landslides induced by heavy rainfall were evaluated by comparing two landslides in artificial forests of different ages (Kake and Asakura). We focused on rainfall characteristics that trigger landslides and the driftwood volumes produced by the resulting landslides. Rainfall characteristics that triggered landslides were estimated using a three-layer tank model and normalized according to the RP. The results for the two events and the driftwood volume per unit catchment area were compared. At Kake and Asakura, the RP of the first tank storage layer, which corresponded to the temporal variation in groundwater level in the shallow soil layer, affected the occurrence of shallow landslides. However, this value was 3.0-fold higher for landslides at Asakura than for those at Kake, due to the higher threshold protecting against shallow landslides in mature than in young forests. Driftwood volume produced by landslides was 30-fold greater at Asakura than at Kake, and the damage was accordingly higher. Therefore, our findings indicate that forests develop increased resistance to shallow landslides as they mature. However, when heavy rainfall exceeds this resistance, damage from sediment-related disasters may be significant due to the large volumes of driftwood produced. By advancing our understanding of the influence of forest cover changes on rainfall-induced shallow landslides, our findings will promote the development of more effective landslide risk management strategies.

## Data Availability

Rainfall data for Kake and Asakura are available from the Japanese Meteorological Agency website: (https://www.data.jma.go.jp/gmd/risk/obsdl/index.php).
